# Evaluation of monkeypox knowledge and attitudes among Chinese medical students

**DOI:** 10.1186/s12889-024-18259-6

**Published:** 2024-03-08

**Authors:** Liliang Yu, Yan Shen, Min Liu, Junchun Ma, Jiang Long, Daikun Zheng

**Affiliations:** 1 Chongqing Three Gorges Medical College, Chongqing, China; 2 Wanzhou District Center for Disease Control and Prevention, Chongqing, China; 3 Chongqing Center for Disease Control and Prevention, Chongqing, China

**Keywords:** Monkeypox, Knowledge, Attitude, Chinese medical students

## Abstract

**Background:**

Monkeypox is a zoonotic disease caused by the monkeypox virus and is increasingly recognized as a serious public health concern worldwide. Our aim was to investigate the knowledge and attitudes of Chinese medical students regarding monkeypox.

**Methods:**

A cross-sectional study was conducted among 8,897 college students from China. An e-questionnaire was used to collect data on knowledge (17 items), attitudes (12 items), and baseline criteria. The relationships between a range of factors and knowledge and attitudes were studied using univariate and multivariate analyses.

**Results:**

A total of 79.33% of the study participants were female, 89.10% were of Han ethnicity, 72.50% were from rural areas, 50.39% were in their first year, and 80.65% were medical majors. A total of 50.88% had good knowledge of monkeypox, and 57.11% had a positive attitude towards monkeypox knowledge. Univariate analysis revealed that origin and major were the factors affecting the knowledge level of monkeypox among participants. Rural students had more knowledge of monkeypox than urban students, and nonmedical students had greater awareness of monkeypox than did medical students. Moreover, sex and grade were the factors influencing participants’ attitudes towards monkeypox; men had more positive attitudes than women did, and senior students had more positive attitudes than junior students did. Multivariate analysis revealed that major and the origin of the students independently influenced the monkeypox knowledge of Chinese medical students, while sex, grade and monkeypox knowledge were significantly related to attitudes towards monkeypox.

**Conclusion:**

This study revealed that nearly half of the Chinese medical students had good knowledge and a positive attitude towards monkeypox. Student origin and major independently influenced the knowledge of Chinese medical students of monkeypox, while sex, grade and knowledge were independently related to the attitudes of Chinese medical students towards monkeypox.

**Supplementary Information:**

The online version contains supplementary material available at 10.1186/s12889-024-18259-6.

## Introduction

Monkeypox is a zoonotic infectious disease characterized by fever, rash, and lymph node enlargement caused by infection with the monkeypox virus [1–4]. It is one of the four recognized smallpox virus infections after the eradication of smallpox. The virus was first discovered in monkeys in 1958, and monkeypox virus was first isolated from a specimen of a suspected smallpox patient in the Democratic Republic of Congo in 1970; this case was the first confirmed case of human monkeypox [5, 6]. Before 2021, monkeypox was mainly endemic in Central and West Africa [7, 8] and was mainly transmitted through contact with infected animals, resulting in a short chain of human-to-human transmission and occasional spread to other countries and regions through family or travel. However, since May 2022, multiple countries worldwide have experienced outbreaks of monkeypox [9–14], and in July 2022, the World Health Organization declared the disease a public health emergency of international concern [15]. In addition, according to the literature, the mortality rate associated with monkeypox disease is as high as 10% [16]. Due to the high infectivity and mortality rate of monkeypox, timely measures are urgently needed to contain the epidemic.

As one of the most densely populated and complex places, universities have the potential to become venues for monkeypox transmission. As a special group, college students’ cognition, attitudes, and behaviours towards diseases not only determine their physical and mental health but also have an impact on the knowledge, attitudes, and behaviours of the surrounding population. A cross-sectional study [17] involving 314 Saudi Arabian university students showed that the vast majority of medical students (72%) had poor knowledge of the monkeypox virus. Kumar et al. [18] conducted a cross-sectional survey on the knowledge, attitudes, behaviours, and willingness to vaccinate against monkeypox among 946 Pakistani university students and revealed that only 6.3% of the students had good knowledge of monkeypox and that only 20.5% had a positive attitude towards monkeypox. In addition, the study revealed significant correlations between monkeypox knowledge and students’ educational background, major, and region. These studies indicate that even though the current situation of monkeypox outbreaks abroad is severe, local college students still lack abundant knowledge and a positive attitude towards monkeypox, which may be one of the reasons why the foreign epidemic has not been effectively controlled.

Monkeypox disease is recognized as a national Class B management infectious disease in China. According to data from the China Center for Disease Control and Prevention, in August 2023, China reported 501 new confirmed cases of monkeypox, indicating that the monkeypox epidemic situation in China is not optimistic. To more precisely curb the development of the monkeypox epidemic, a cross-sectional survey was conducted among college students, aiming to clarify the current status of their knowledge and attitudes related to monkeypox and analyse the influencing factors, identify existing problems and weak links in epidemic prevention and control, and provide an important reference for universities to perform well in epidemic prevention and control work and relevant departments to develop precise prevention and control measures.

## Methods

### Study setting and duration

This was a cross-sectional online questionnaire survey involving students in three different grades at a public college in China. This public college, located in southern China, comprises 6 academies in a range of academic disciplines, including medicine, bioengineering, and computer science. Most of the students in this school are engaged in basic medical care, which makes our study area suitable for conducting research on such emerging public health issues.

The study period was from September 19, 2023, to November 1, 2023. We followed the STRE Guide during our research. Ethical approval was obtained from Chongqing Three Gorges Medical College of Science and Technology before the initiation of this study, and informed consent was obtained from all participants before the start of the investigation. The participants’ information was not disclosed.

After a careful quality review, a total of 8897 questionnaires were included in the research analysis.

### Questionnaire

The e-questionnaire was modified and developed after expert discussion following an in-depth review of the relevant literature [19, 20]. The questionnaire was divided into three parts: the first part collected sociodemographic information about the participants, such as their sex, ethnic group, origin, grade and professional category; the second part included 17 questions assessing the students’ knowledge of monkeypox disease regarding its nature, transmission route, clinical performance and management; and the third part evaluated participants’ attitudes towards monkeypox infection using 12 questions, each with five options ranging from completely disagree to completely agree. The answers to the knowledge item (17 questions) were assigned as follows: “yes” was assigned 2 points, “uncertain” was assigned 1 point, and “no” was assigned 0 points. The highest score in the knowledge section was 34 points, and the higher the score was, the better the knowledge of monkeypox was. The answers to the attitude item (12 questions) were as follows: “strongly agree”, 5 points; “agree”, 4 points; “neutral”, 3 points; “disagree”, 2 points; and “strongly disagree”, 1 point. The highest possible score for attitude-related questions was 60 points, with a higher score indicating a more positive attitude towards monkeypox.

First, a small-scale survey (81 copies) was conducted. The data showed that the Cronbach’s α coefficients of knowledge and attitude were 0.734 and 0.781, respectively, indicating good internal consistency of the questionnaire. We subsequently conducted a formal survey, and after the questionnaire was collected, our investigators checked the completeness, internal consistency and rationality of the questionnaire. Before the statistical analysis, we conducted strict quality control on the questionnaire to obtain an effective questionnaire. The data deletion criteria were as follows: (1) incomplete data; (2) data anomalies, including the selection of all the same options and the selection of options in order or not logically; and (3) a less than 90-second answer time. After a careful quality review, a total of 8897 questionnaires were included in the research analysis.

### Statistical analyses

Raosoft’s sample size calculator was used to calculate the minimum sample size. The confidence level was 95%, and the acceptable margin of error was 5%. The response rate was expected to reach 50%. As of September 2023, the number of college students in China was 44.3 million. Therefore, the sample size n was calculated as follows: *x* = *Z*(^*c*^/100) ^2^*r*(100-*r*), *n* = ^*N x*/^(*N*-1)*E*^2^ + *x*), where N is the population size, r is the fraction of the response, and Z(c/100) is the critical value of confidence level c [21]. Moreover, we expected this study to have an invalid questionnaire rate of 20% and ultimately calculated the minimum sample size needed to include at least 482 individuals.

The data were analysed using IBM SPSS Statistics v25.0. After Kolmogorov-Smirnov test (*P* < 0.05), combined with histogram and Q-Q plot (Additional file: Fig S1-S4), knowledge and attitude scores do not obey normal distribution. Continuous data are presented as medians and interquartile ranges (Ms and IQRs), and categorical data are expressed as frequencies and percentages (N, %). Before performing the exploratory factor analysis, factorability was assessed using both the Kaiser–Meyer–Olkin (KMO) test and Bartlett’s test of sphericity. Knowledge and attitude scores were classified according to the median. Participants who scored 28 points above the median were considered to have good knowledge, while those who scored less than or equal to the median were considered to have poor knowledge. Similarly, participants who scored above the median value of 46 were considered to have a positive attitude, while those who scored below or equal to the median were considered to have a negative attitude. Differential analysis was performed using the chi-square test, and a logistic regression model was used for multivariate analysis. *P* < 0.05 was considered to indicate statistical significance.

## Results

### Basic characteristics of the respondents

Table 1 presents the basic characteristics of the 8,897 college students included in this questionnaire survey. The main students who participated in the questionnaire survey were female (79.33%) and were of Han ethnicity (89.10%), and more students came from rural areas (72.50%). Of the students who participated in the survey, 50.39% were in their freshman year, 30.71% were in their sophomore year, and 18.91% were in their junior year. Among the respondents, 80.65% were medical majors, and the others were nonmedical majors (19.35%).

**Table 1:** Basic characteristics of the respondents

Characteristics	n	%
**Sex**		
Male	1839	20.67
Female	7058	79.33
**Ethnicity**		
Han	7927	89.10
Others	970	10.90
**Origin of student**		
City	2447	27.50
Rural	6450	72.50
**School year**		
First year	4483	50.39
Second year	2732	30.71
Third year	1682	18.91
**Major**		
Medicine	7175	80.65
Others	1722	19.35

### Knowledge about monkeypox

The Cronbach’s α of monkeypox knowledge was 0.734, the KMO was 0.822, and the Bartlett’s test of sphericity *P* < 0.001 indicated that the scale had good reliability and validity. The median monkeypox knowledge score was 28 (25, 32). Participants had good knowledge of the nature, management, and clinical manifestations of monkeypox disease. However, 32.20% of the students were still unsure whether the monkeypox vaccine was available, 26.45% were unsure that monkeypox could be transmitted through food, and 22.70% were uncertain that monkeypox infection could be prevented by wearing condoms during sexual activity (Table 2). Overall, 50.88% of the participants had good knowledge of monkeypox.

**Table 2:** Knowledge of medical students about monkeypox (*N* = 8897)

Components of knowledge scale	Yes (n, %)	Uncertain (n, %)	No (n, %)
1. Monkeypox is a viral infectious disease.	8735 (98.18)	99 (1.11)	63 (0.71)
2. Monkeypox, like COVID-19, is a national class B management infectious disease.	7716 (86.73)	356 (4.00)	825 (9.27)
3. Monkeypox is a zoonotic disease.	7295 (81.99)	535 (6.01)	1067 (11.99)
4. The incubation period of monkeypox is usually 6$\sim$16 days, but it can also be 5$\sim$21 days.	7398 (83.15)	258 (2.90)	1241 (13.95)
5. Monkeypox infection requires isolation.	8357 (93.93)	194 (2.18)	346 (3.89)
6. A monkeypox vaccine has been developed.	3107 (34.90)	2925 (32.90)	2865 (32.20)
7. Monkeypox can be transmitted through food.	4442 (49.93)	2102 (23.63)	2353 (26.45)
8. Monkeypox can be transmitted through blood.	7043 (79.16)	463 (5.20)	1391 (15.63)
9. Monkeypox can be transmitted sexually, including among men who have sex with men.	6930 (77.89)	482 (5.42)	1485 (16.69)
10. Monkeypox can be transmitted from animals to humans through direct contact.	5987 (67.29)	1109 (12.46)	1801 (20.24)
11. Rash is one of the clinical manifestations of monkeypox.	7293 (81.97)	309 (3.47)	1295 (14.56)
12. Swollen lymph nodes distinguish monkeypox from chickenpox.	6339 (71.25)	430 (4.83)	2128 (23.92)
13. Avoiding contact with wild animals is an important way to prevent the further spread of monkeypox.	7280 (81.83)	520 (5.84)	1097 (12.33)
14. Avoiding contact with anyone with a rash can prevent the spread of the disease.	5959 (66.98)	1865 (20.96)	1073 (12.06)
15. Avoiding contact with any objects that have come into contact with sick people can prevent the spread of the disease.	6909 (77.66)	1073 (12.06)	915 (10.28)
16. Wearing condoms during sex is effective in preventing monkeypox infection.	4121 (46.30)	2752 (30.90)	2024 (22.70)
17. Improving monkeypox awareness and awareness of prevention and control and self-symptom monitoring are key measures to prevent monkeypox.	8262 (92.86)	115 (1.29)	520 (5.84)
Knowledge Score, M (IQR)	28 (25, 32)		

### Attitude towards monkeypox

The Cronbach’s α of the attitude scale was 0.781, the KMO was 0.865, and the Bartlett’s test of sphericity *P* < 0.001 indicated that the scale had good reliability and validity. Overall, 57.11% of participants had a positive attitude towards monkeypox, with a median attitude score of 46 (42, 49). A total of 59.68% of the students strongly agreed that “I should know more about monkeypox disease”; 59.32% of the students strongly agreed that “I should take some preventive measures to prevent the occurrence of monkeypox”; 58.27% of the students strongly agreed that “the government should fully provide monkeypox prevention and control measures to local residents”; 52.52% of the students strongly agreed that “if there is a monkeypox vaccine, I will choose to get vaccinated”; and nearly half of the students strongly agreed that “medical workers should test for infection after contact with monkeypox patients”. Conversely, approximately one-third of the students strongly disagreed that “I can reach out to a family member or friend infected with monkeypox” (Table 3).

**Table 3:** Attitudes of medical students about monkeypox (*n* = 8897)

Components of attitude scale	Strongly agree	Agree	Neutral	Disagree	Strongly disagree
1. I should know more about monkeypox.	5310 (59.68)	2459 (27.64)	598 (6.72)	40 (0.45)	490 (5.51)
2. The spread of the monkeypox virus to my city worries me.	2696 (30.30)	3587 (40.32)	1972 (22.16)	324 (3.64)	318 (3.57)
3. The government should provide adequate monkeypox prevention and control measures to local residents.	5184 (58.27)	2921 (32.83)	613 (6.89)	26 (0.29)	153 (1.72)
4. Travel to monkeypox-infected countries should be restricted.	3369 (37.87)	3207 (36.05)	1770 (19.89)	345 (3.88)	206 (2.32)
5. If there is a monkeypox vaccine, I will choose to get it.	4673 (52.52)	3413 (38.36)	682 (7.67)	41 (0.46)	88 (0.99)
6. Health care workers should test for infection after contact with monkeypox patients.	4494 (50.51)	3577 (40.20)	642 (7.22)	81 (0.91)	103 (1.16)
7. I can reach out to family members or friends who are infected with monkeypox.	403 (4.50)	625 (7.00)	1622 (18.20)	2840 (31.90)	3407 (38.30)
8. I should take some precautions to prevent monkeypox.	5278 (59.32)	3048 (34.26)	453 (5.09)	29 (0.33)	89 (1.00)
9. All people who develop a rash should be tested for monkeypox.	2418 (27.18)	3327 (37.39)	2387 (26.83)	649 (7.29)	116 (1.30)
10. Monkeypox will become a new pandemic disease like COVID-19.	1244 (13.98)	2105 (23.66)	3319 (37.30)	1685 (18.94)	544 (6.11)
11. Specialized medical institutions provide less information about monkeypox.	922 (10.36)	2424 (27.25)	3913 (43.98)	1158 (13.02)	480 (5.40)
12. Monkeypox may become a disease that reduces the global population.	1052 (11.82)	2352 (26.44)	3542 (39.81)	1531 (17.21)	420 (4.72)
Attitude Score, M (IQR)	46 (42, 49)				

### Associations between baseline characteristics and knowledge and attitudes towards monkeypox

Table 4 shows the relationships between baseline characteristics and monkeypox knowledge and attitudes. Univariate analysis revealed that origin and major affected the knowledge level of monkeypox among participants. Rural students had more knowledge of monkeypox than urban students, and nonmedical students were more aware of this topic than medical students were; however, sex, ethnicity and grade were not significantly related to monkeypox knowledge grouping. Moreover, sex and grade were found to be factors influencing participants’ attitudes towards monkeypox; men had more positive attitudes than women did, and senior students had more positive attitudes than junior students did. However, there were no significant differences in the attitudes towards monkeypox according to ethnicity, place of origin or major.

**Table 4:** Associations between student characteristics and knowledge and attitudes towards monkeypox based on univariate analysis

Baseline characteristics	Knowledge			Attitude		
	Poor	Good	p	Poor	Good	p
**Sex**						
Male	898 (20.55)	941 (20.79)	0.782	700 (18.34)	1139 (22.42)	<0.001^*^
Female	3472 (79.45)	3586 (79.21)		3116 (81.66)	3942 (77.58)	
Ethnicity						
Han	3914 (89.57)	4013 (88.65)	0.164	3412 (89.41)	4515 (88.86)	0.408
Others	456 (10.43)	514 (11.35)		404 (10.59)	566 (11.14)	
**Origin of student**						
City	1273 (29.13)	1174 (25.93)	0.001^*^	1082 (28.35)	1365 (26.86)	0.119
Rural	3097 (70.87)	3353 (74.07)		2734 (71.65)	3716 (73.14)	
**School year**						
First year	2195 (50.23)	2288 (50.54)	0.472	2037 (53.38)	2446 (48.14)	<0.001^*^
Second year	1327 (30.37)	1405 (31.04)		1075 (28.17)	1657 (32.61)	
Third year	848 (19.41)	834 (18.42)		704 (18.45)	978 (19.25)	
**Major**						
Medicine	3577 (81.85)	3598 (79.48)	0.005^*^	3104 (81.34)	4071 (80.12)	0.150
Others	793 (18.15)	929 (20.52)		712 (18.66)	1010 (19.88)	

^*^p < 0.05

### Predictors of knowledge and attitudes towards monkeypox

A multivariate logistic regression model was used to identify the factors influencing students’ knowledge of and attitudes towards monkeypox. As shown in Table 5, students from rural areas were more likely to have better knowledge of monkeypox than students from urban areas were (OR = 1.168, 95% CI = 1.064–1.282). Compared with students in other majors, students majoring in clinical medicine were more likely to have better knowledge of monkeypox (OR = 1.156, 95% CI = 1.040–1.285). With regard to attitudes towards monkeypox, females were 1.307 times (95% CI: 1.175–1.455) more likely to have a positive attitude towards monkeypox than males were, while second-year and third-year college students were less likely to have a positive attitude towards monkeypox than first-year students were (OR = 0.768, 95% CI: 0.696–0.847; OR = 0.849, 95% CI: 0.757–0.953). In addition, students with good knowledge of monkeypox were 1.961 times more likely to have a positive attitude towards monkeypox than were those with poor knowledge of monkeypox (OR = 1.961, 95% CI = 1.8000-2.136).

**Table 5:** Predictors of college students’ knowledge of and attitudes towards monkeypox based on multilevel logistic regression models

Predictors	Knowledge			Attitude	
	OR (95% CI)	P		OR (95% CI)	P
**Sex**					
Male	-	-		1	
Female	-	-		1.307(1.175–1.455)	<0.001^*^
**Origin of student**					
City	1			-	-
Rural	1.168(1.064–1.282)	0.001^*^		-	-
**School year**					
First year	-	-		1	
Second year	-	-		0.768(0.696–0.847)	<0.001^*^
Third year	-	-		0.849(0.757–0.953)	0.005^*^
**Major**					
Others	1			-	-
Medicine	1.156(1.040–1.285)	0.007^*^		-	-
Knowledge					
Poor	-	-		1	
Good	-	-		1.961(1.800-2.136)	<0.001^*^

^*^p < 0.05

## Discussion

This study assessed the knowledge and attitudes of 8897 Chinese medical students regarding monkeypox and identified factors that influenced their knowledge and attitudes towards this topic. Students from rural areas who majored in clinical medicine were more likely to have good knowledge of monkeypox. In terms of attitudes towards monkeypox, women’s attitudes towards monkeypox were positive. First-year students were more likely to be positive about monkeypox than older students were, which may be due to the school’s health education lecture on monkeypox and AIDS for first-year students in September 2023. In addition, monkeypox knowledge was positively associated with positive attitudes towards monkeypox. The analysis results of this study showed that the mastery and attitudes of Chinese medical students regarding monkeypox knowledge were not ideal, and the proportions of students with good knowledge of monkeypox and positive attitudes towards monkeypox were 50.88% and 57.11%, respectively. Studies have shown that the knowledge of monkeypox among doctors and nurses is 55% and 57.97% [22, 23], respectively, indicating that the knowledge of monkeypox among medical students is lower than that among doctors and nurses. This finding is consistent with previous findings. A study [20] evaluating the knowledge and attitudes of medical students from 27 countries on three continents showed that 55.3% of the students had good knowledge of monkeypox and that 51.7% of the medical students had positive attitudes towards monkeypox. However, another study [18] among 946 Pakistani medical students showed that only 6.3% of students had good knowledge of monkeypox, and 20.5% had a positive attitude towards monkeypox, which may have been closely related to the type of education and specialty of the respondents. The above studies show that current medical students have relatively little knowledge of monkeypox and lack enthusiasm for gaining this knowledge.

The situation of the monkeypox outbreak in China is also not optimistic. A study [24] based on a community in Shenzhen, China, showed that only approximately half of the general population had a high awareness of monkeypox (56.5%) and related symptoms (49.7%), and only approximately one-third (37.1%) expressed high concern about monkeypox. The lack of adequate awareness and positive attitudes among citizens regarding monkeypox may also explain why the monkeypox outbreak has not been effectively controlled. It is necessary to implement highly effective and targeted measures to contain the monkeypox outbreak, especially for college students. Our study fills the gap in the knowledge and understanding of the attitudes of domestic college students towards monkeypox and clarifies the relevant influencing factors of these to provide a direction for targeted interventions to curb the spread of monkeypox in China.

### Strengths and limitations of this study

This study is the first to investigate the knowledge and attitudes of Chinese medical students and identify the factors affecting monkeypox knowledge and attitudes, providing a theoretical basis for formulating effective strategies for preventing and controlling monkeypox epidemics at the school level in China. In addition, the research population was composed mainly of medical students, who are the main source of primary medical and health care personnel in China in the future. This information is highly important for effectively improving the ability of grassroots doctors to respond to health emergencies and reduce harm from public health emergencies. However, this study has the following limitations. First, the questionnaire respondents were mainly from a public university in Chongqing, and there was a certain selection bias. However, college students come from all over the country, and they also work all over the country after graduation, which to a certain extent represents the current situation of the knowledge and attitudes of college students towards monkeypox across the country. Second, the research data relied on self-reports from respondents, and there may have been some information bias. However, the questionnaires we collected were carefully reviewed to ensure the accuracy of the findings. Finally, in September 2023, this medical college conducted a lecture on monkeypox and AIDS for first-year students; our survey revealed that first-year students had more positive attitudes towards monkeypox than senior students did, which also provides theoretical support for follow-up health education for college students.

## Conclusions

This study revealed that nearly half of the Chinese medical students had good knowledge and a positive attitude towards monkeypox. Student origin and major independently influenced the knowledge of Chinese medical students of monkeypox, while sex, grade and knowledge were independently related to the attitudes of Chinese medical students towards monkeypox.

### Electronic supplementary material

Below is the link to the electronic supplementary material.

Supplementary Material 1

Supplementary Material 2

Supplementary Material 3

Supplementary Material 4

Supplementary Material 5

Supplementary Material 6

## Data Availability

The datasets used and/or analyzed during this survey are available from the corresponding author upon reasonable request.
