# Supplementary figures and images for: Evaluation of monkeypox knowledge and attitudes among Chinese medical students
Source: BMC Public Health. 2024 Mar 8;24:745. doi: 10.1186/s12889-024-18259-6 (PMC10921768; doi:10.1186/s12889-024-18259-6)

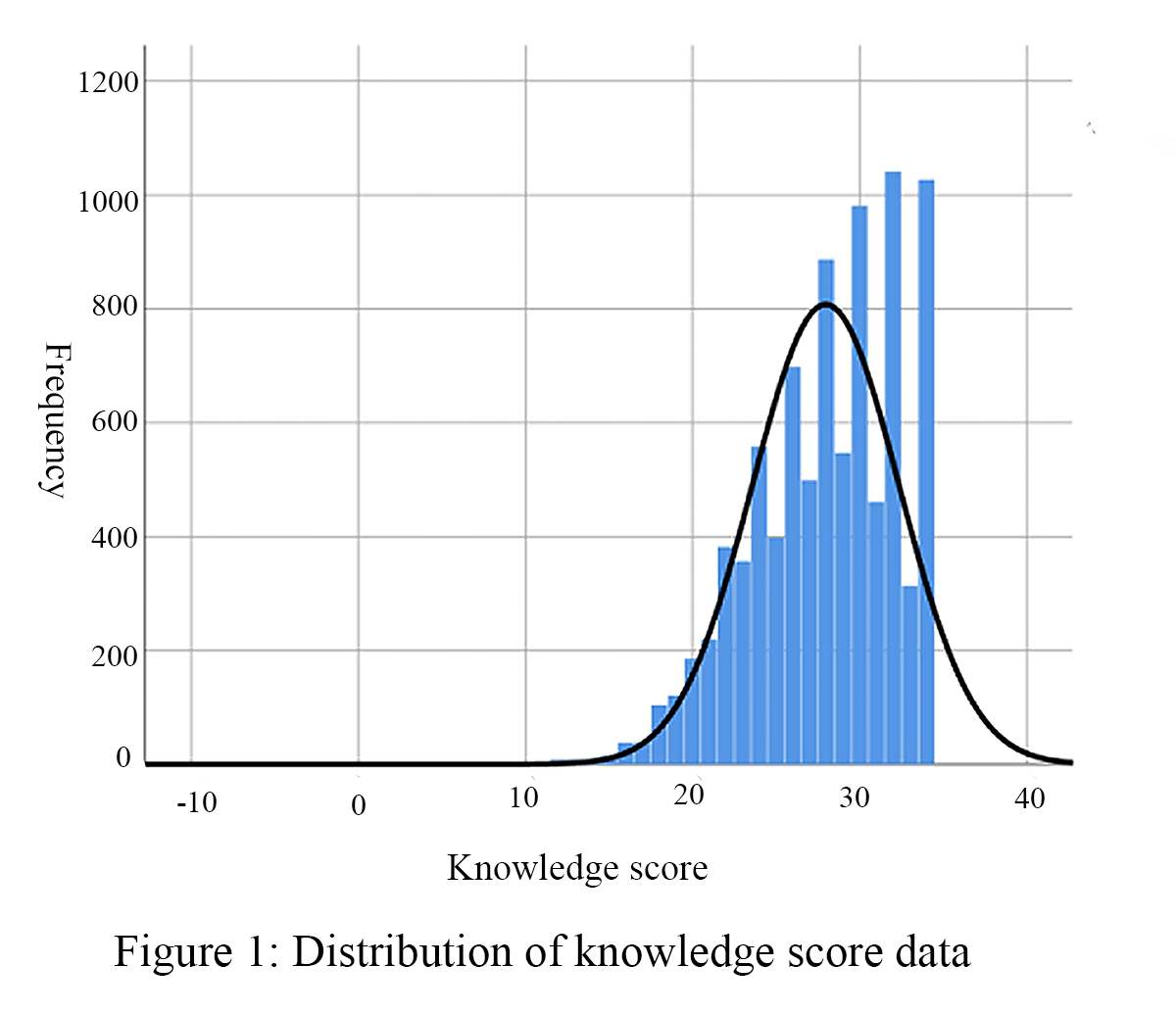

Supplement: Supplementary file 3 — Supplementary Material 3 [file 12889_2024_18259_MOESM3_ESM.tif]

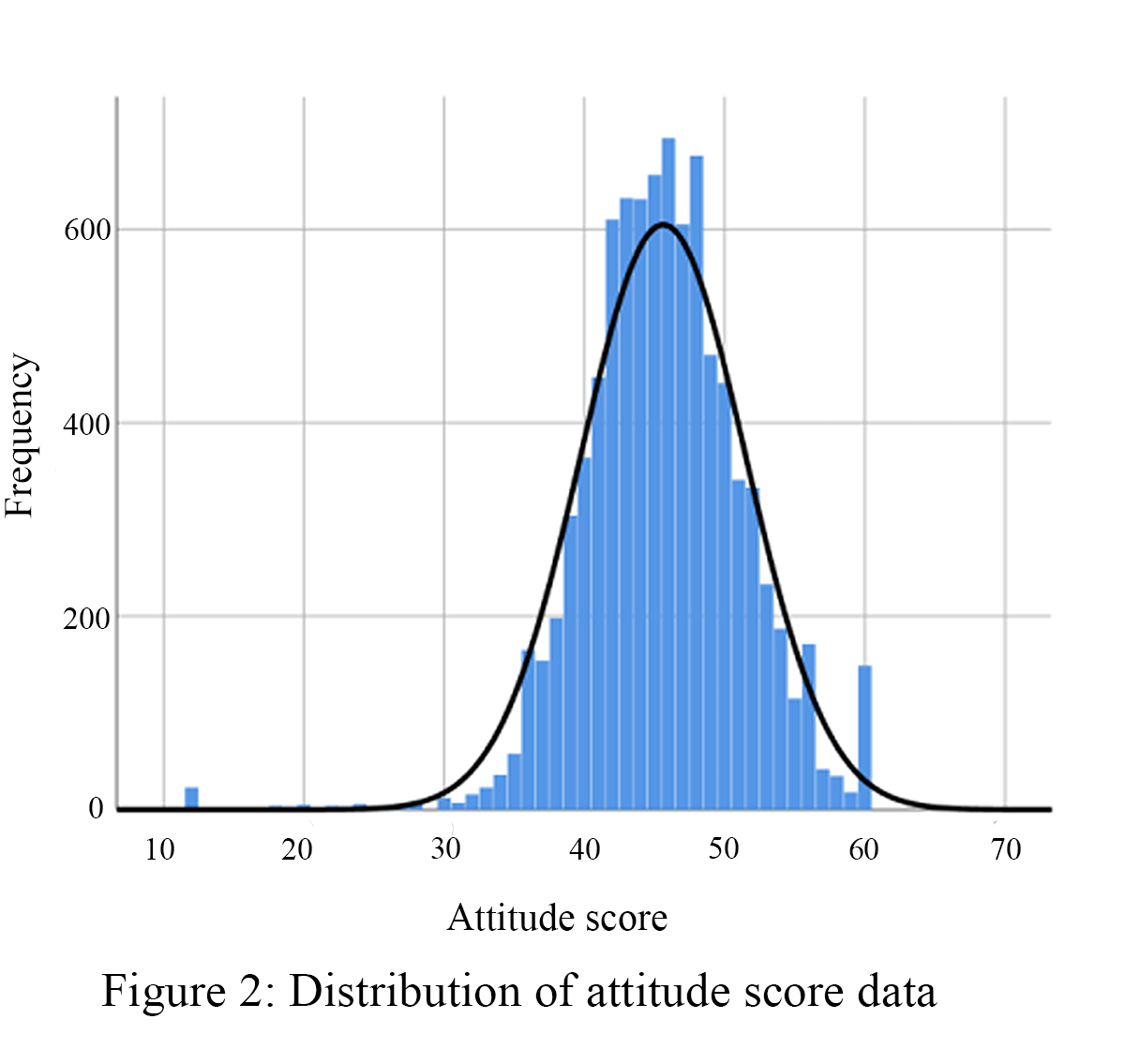

Supplement: Supplementary file 4 — Supplementary Material 4 [file 12889_2024_18259_MOESM4_ESM.tif]

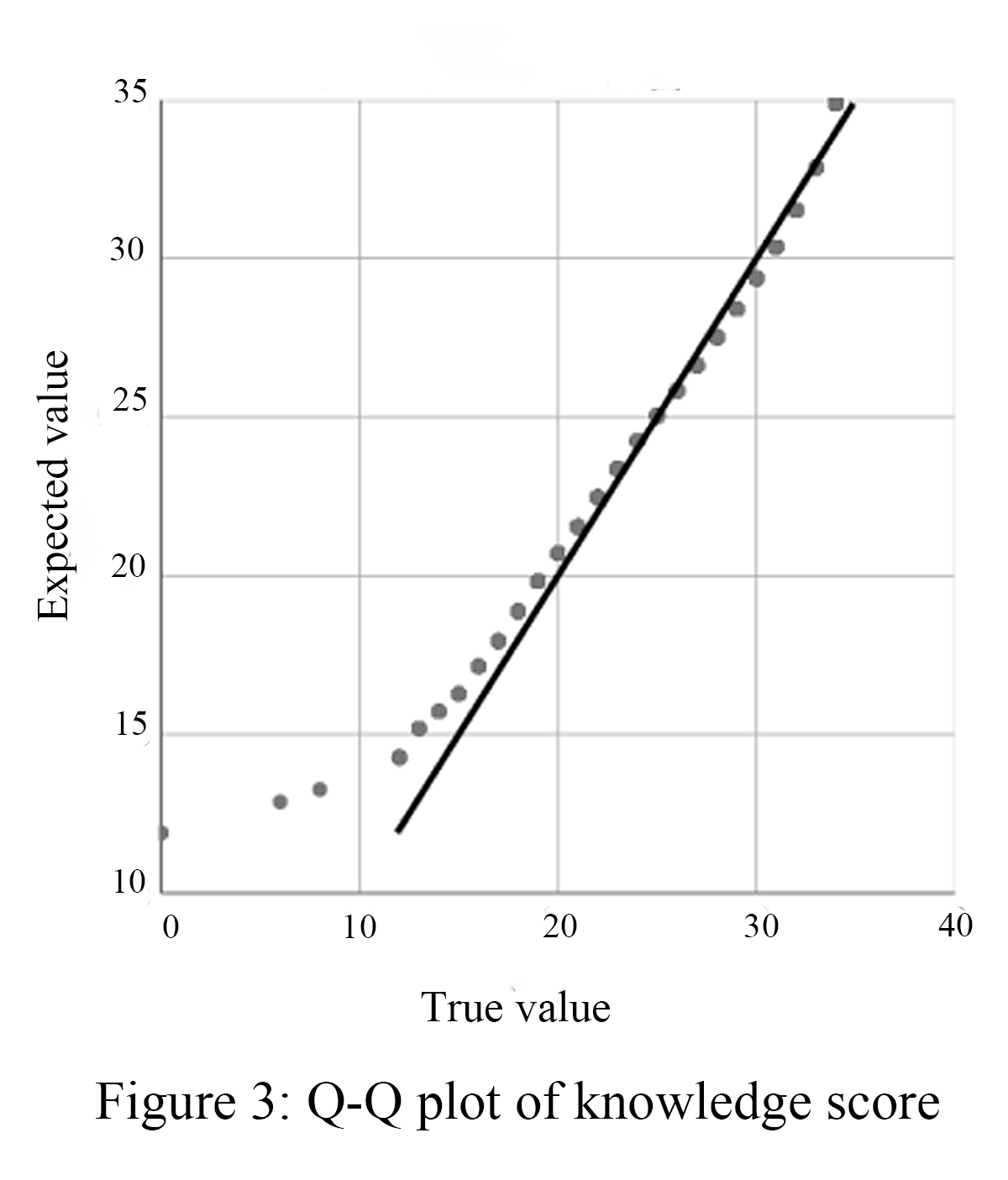

Supplement: Supplementary file 5 — Supplementary Material 5 [file 12889_2024_18259_MOESM5_ESM.tif]

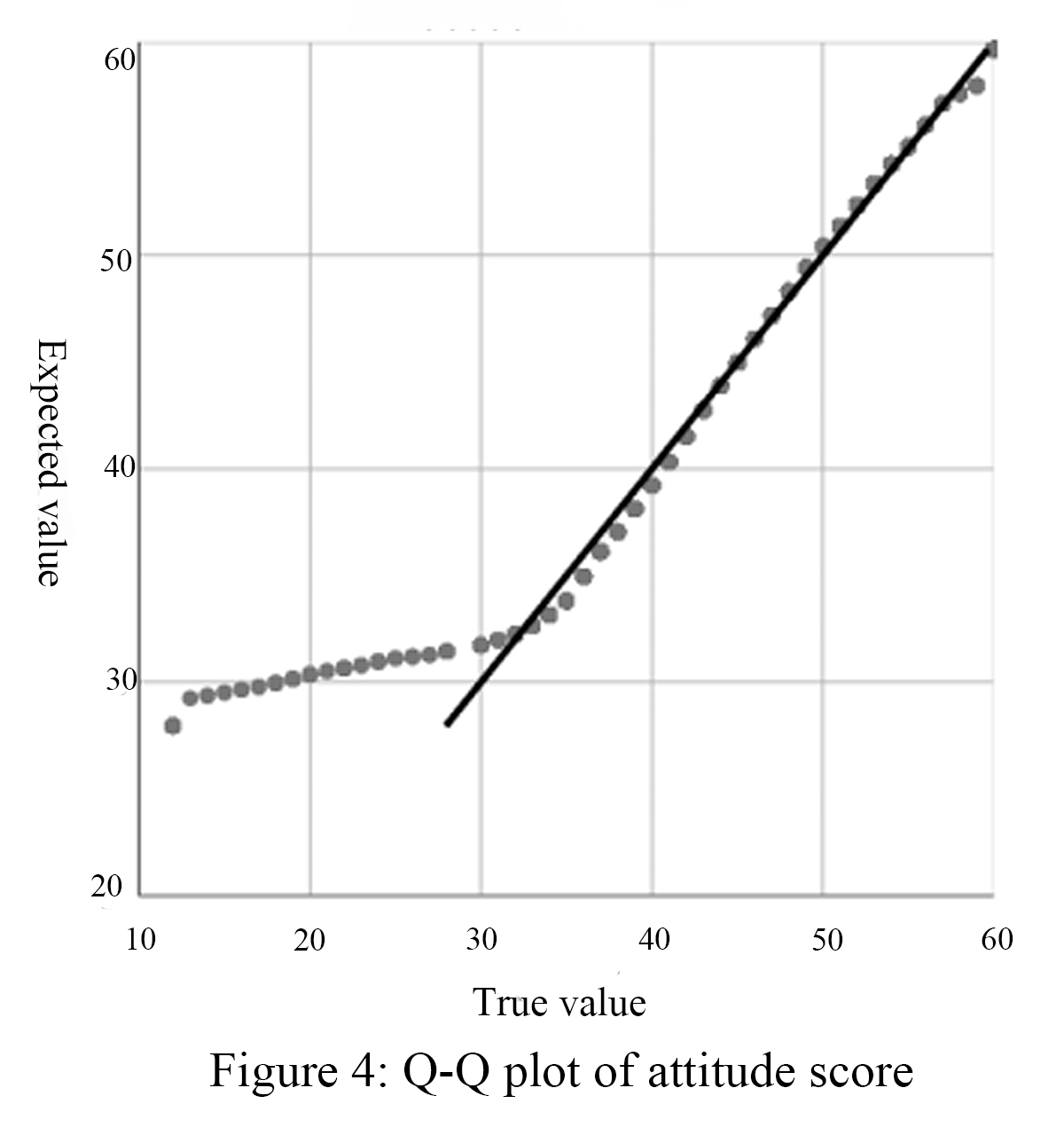

Supplement: Supplementary file 6 — Supplementary Material 6 [file 12889_2024_18259_MOESM6_ESM.tif]
